# MPV17L2 is required for ribosome assembly in mitochondria

**DOI:** 10.1093/nar/gku513

**Published:** 2014-06-19

**Authors:** Ilaria Dalla Rosa, Romina Durigon, Sarah F. Pearce, Joanna Rorbach, Elizabeth M.A. Hirst, Sara Vidoni, Aurelio Reyes, Gloria Brea-Calvo, Michal Minczuk, Michael W. Woellhaf, Johannes M. Herrmann, Martijn A. Huynen, Ian J. Holt, Antonella Spinazzola

**Affiliations:** 1 MRC National Institute for Medical Research, Mill Hill, London NW7 1AA, UK; 2 MRC Mitochondrial Biology Unit, Wellcome Trust-MRC Building, Hills Road, Cambridge CB2 0XY, UK; 3 Cell Biology, University of Kaiserslautern, 67663 Kaiserslautern, Germany; 4 Centre for Molecular and Biomolecular Informatics, Radboud University Medical Centre, Geert Grooteplein Zuid 26–28, 6525 GA Nijmegen, Netherlands

## Abstract

MPV17 is a mitochondrial protein of unknown function, and mutations in *MPV17* are associated with mitochondrial deoxyribonucleic acid (DNA) maintenance disorders. Here we investigated its most similar relative, MPV17L2, which is also annotated as a mitochondrial protein. Mitochondrial fractionation analyses demonstrate MPV17L2 is an integral inner membrane protein, like MPV17. However, unlike MPV17, MPV17L2 is dependent on mitochondrial DNA, as it is absent from ρ^0^ cells, and co-sediments on sucrose gradients with the large subunit of the mitochondrial ribosome and the monosome. Gene silencing of *MPV17L2* results in marked decreases in the monosome and both subunits of the mitochondrial ribosome, leading to impaired protein synthesis in the mitochondria. Depletion of MPV17L2 also induces mitochondrial DNA aggregation. The DNA and ribosome phenotypes are linked, as in the absence of MPV17L2 proteins of the small subunit of the mitochondrial ribosome are trapped in the enlarged nucleoids, in contrast to a component of the large subunit. These findings suggest MPV17L2 contributes to the biogenesis of the mitochondrial ribosome, uniting the two subunits to create the translationally competent monosome, and provide evidence that assembly of the small subunit of the mitochondrial ribosome occurs at the nucleoid.

## INTRODUCTION

The mammalian mitochondrial proteome comprises 1500 or more gene products. The deoxyribonucleic acid (DNA) inside mitochondria DNA (mtDNA) contributes only 13 of these proteins, and they make up ∼20% of the subunits of the oxidative phosphorylation (OXPHOS) system, which produces much of the cells energy. All the other proteins of the organelle are nuclear encoded, synthesized in the cytosol and then imported into the mitochondria. A substantial number of these proteins have roles associated with the structure, production and maintenance of the respiratory chain and adenosine triphosphate synthase, being structural components or assembly factors thereof, or contributors to mtDNA maintenance and expression. However, the precise role of many mitochondrial proteins remains unknown, limiting our understanding of the organelle's role in physiological and disease processes.

The construction of a mitochondrial proteome database comprising over 1000 proteins has facilitated the discovery of mitochondrial disease-genes, such as *MPV17* (1). In 2006, the MPV17 protein, previously assigned as having peroxisomal localization (2), was predicted instead to be a mitochondrial protein (3) and then experimentally shown to localize exclusively to the inner membrane of mitochondria (3). In the latter study, MPV17 dysfunction was also linked to a form of mitochondrial DNA depletion syndrome (3), and later with multiple deletions of mtDNA (4,5). However, neither the function of the MPV17 protein, nor the mechanism leading to mtDNA perturbation is currently known.

In mammals, MPV17 is homologous to three other proteins: MPV17-like protein (MPV17L), MPV17-like 2 protein (MPV17L2 or FKSG24) and peroxisomal membrane protein 2 (PXMP2). Existing literature suggest a peroxisomal localization for PXMP2p (6,7) and dual localization of MPV17L in mitochondria and peroxisomes (8,9). A recent study proposes that PXMP2 forms a constitutively open pore within the peroxisomal membrane, which is voltage-independent and displays weak cationic selectivity (10). Hitherto, nothing was known about the function of MPV17L2. However, previous studies of the mitochondrial proteome have assigned it as a mitochondrial protein based on Bayesian integration of genomics data (1) and a green fluorescent protein (GFP) tagged version of the protein is targeted to the mitochondria (11).

Here we resolve the phylogenetic relationships of the four mammalian MPV17-related proteins, and report a first characterization of the homologue most similar to MPV17, namely MPV17L2. We show that MPV17L2 is an inner mitochondrial membrane protein that is associated with mitochondrial nucleic acids. Specifically, MPV17L2 interacts with the large subunit of the mitochondrial ribosome and the monosome, and when its expression is reduced by ribonucleic acid (RNA) interference, the ribosome is disrupted and translation in the mitochondria is impaired, indicating MPV17L2 plays an important role in ribosomal biogenesis in the organelle.

## MATERIALS AND METHODS

### Plasmid preparation

Human complementary DNA (cDNA) specifying *MPV17* (IMAGE: 5217853) was introduced into Flp-In T-REx human embryonic kidney cells (HEK293T, Life Technologies) to establish inducible, transgenic cell lines. The transgene carried a carboxy-terminal linker sequence followed by octapeptide (DYKDDDDK) (FLAG) and StrepII tags.

### Cell culture and transfection

HEK293T cells were grown in Dulbecco's Modified Eagle's Medium (Life Technologies) supplemented with 10% fetal bovine serum (Fetal bovine serum (FBS), Hyclone) 1% penicillin and streptomycin (PS, Life Technologies), 15 μg/ml Blasticidin^S^ and 100 μg/ml Zeocin (Biosciences). For the generation of inducible transgenic MPV17 FLAG-StrepII cell lines, transfection was mediated using Lipofectamine 2000 (Life Technologies) according to manufacturer's guidelines. Following transfection, cells underwent selection in DMEM supplemented with 10% tetracycline-free FBS (Biochrom), 1% PS, 15 μg/ml Blasticidin^S^ and 100 μg/ml hygromycin^B^ (Sigma). Gene expression was induced by adding doxycycline (Sigma) to the culture medium with a final concentration of 10 ng/ml for 24 h. HEK293T ρ^0^ cells were generated as previously described for avian cells (12). HeLa cells were cultured in DMEM supplemented with 10% FBS, 1% PS and 50 μg/ml uridine. For transient depletion of mtDNA, parental HEK293Tcells were cultured in standard media described above supplemented with 100 ng/ml ethidium bromide for 96 h.

### RNA interference of *MPV17L2*

Reverse transfection knockdown experiments were performed using 100 000 HeLa cells in antibiotic-free media containing 50 μg/ml uridine, using either siRNA targeting *MPV17L2* (Ambion Silencer Select A2: s392421, 5′-CUGCACU ACUGGUACUUGU-3′ A3: s39422, 5′-CCCATGAAGATGGATGATCA-3′, or Origene O1: 5′-UGAUCAUCCAUCUUCAUGG-3′) or an unrelated non-targeting siRNA (Ambion Silencer select siRNA negative control 4390846) at 10 nM delivered using Lipofectamine RNAiMax (Life Technologies) according to manufacturer's specifications. HeLa cells underwent either one or two rounds of siRNA treatment, with the second transfection performed 72 h after the initial transfection. After 6 days, cells were harvested and processed for quantitative real-time PCR (Q-PCR), immunoblotting, sucrose gradient, immunofluorescence or mitochondrial translation analysis.

### Immunofluorescence and electron microscopy

HeLa cells grown on glass coverslips were treated with 100 nM MitoTracker^®^ Red for 30 min, fixed with 4% paraformaldehyde in phosphate-buffered saline (PBS) and permeabilized with 0.3% Triton X-100 in PBS containing 5% FBS. After permeabilization, samples were blocked with 5% FBS in PBS and incubated with the indicated primary antibodies: anti-DNA (dilution 1:150; Progen), MRPS27 (dilution 1:200, Proteintech), MRPS18 (dilution 1:200, Proteintech), MRPL45 (dilution 1:200, Proteintech). After three washes, slides were incubated with the following secondary antibody: AlexaFluor^®^-488 goat–anti-mouse antibodies or AlexaFluor^®^ 568 donkey anti-rabbit (dilution: 1:1000; Molecular Probes). Finally, cover slips were washed with PBS and mounted on glass slides by inversion over ProLong^®^ Gold Antifade Reagent that includes 4',6-diamidino-2-phenylindole (DAPI) nuclear stain. In preparation for electron microscopy HeLa cells were fixed in 2% glutaraldehyde/2% paraformaldehyde for 30 min, and then incubated for 1 h with 1% osmium tetroxide using 0.1 M sodium cacodylate buffer pH 7.2, at room temperature. The samples were then dehydrated and embedded in Epon resin. Sections were stained with ethanolic uranyl acetate and Reynold's lead citrate, and viewed with a Jeol 100EX Transmission electron microscopy (TEM). Images were captured with a Gatan Orius 1000 Charge-coupled devices (CCD).

### Mitochondrial isolation, protease protection assay, carbonate extraction and iodixanol gradient fractionation

Mitochondria were isolated as described (13). Briefly, HEK293T cells were disrupted by homogenization in hypotonic buffer (20 mM HEPES pH 8, 5 mM KCl, 1.5 mM MgCl_2_ and 2 mM DTT), and mixed with a mannitol–sucrose buffer to final concentrations of 210 mM mannitol, 70 mM sucrose, 20 mM HEPES pH 8 and 2 mM EDTA (1 × MSH), prior to purification of mitochondria by differential centrifugation. The protease protection assay was modified from (14). Briefly, 50 μg aliquots of mitochondria were resuspended in 0.1 ml of hypotonic buffer (20 mM HEPES-OH pH 7.6) in the presence or absence of 5 μg/ml of trypsin and incubated at 30°C for the indicated times. Trypsin digestion was quenched by the addition of 1.5 mg/ml SBTI (Soya bean Trypsin Inhibitor, Sigma) and further incubation on ice for 10 min. Mitochondria from all samples were then isolated by centrifugation, and washed twice with MSH buffer before resuspension with sodium dodecyl sulphate-polyacrylamide gel electrophoresis (SDS-PAGE) loading buffer. Sensitivity of proteins to trypsin digestion was assayed with SDS-PAGE followed by immunoblotting with the indicated antibodies.

For carbonate extraction of mitochondrial proteins, 2 mg of HEK293T mitochondria were treated with either 0 or 100 mM sodium carbonate pH 11.5. A fraction of the mitochondria were treated additionally with 0.1 or 1% sodium deoxycholate for 30 min on ice/4°C. All samples were then centrifuged at 122 000 *g* for 30 min and the supernatant and pellet fractions were analysed by immunoblotting, after trichloroacetic acid (TCA) precipitation.

For iodixanol gradient fractionation, 1000 g supernatant from lysed mitochondria was loaded onto 20–42.5% discontinuous iodixanol (Optiprep, Sigma) gradients (Gradient buffer: 20 mM HEPES pH 7.8, 1 mM EDTA, 50 mM NaCl, 2 mM DTT, 0.05% DDM with 1:50 (v/v) Roche protease inhibitor) and centrifuged at 100 000 *g* for 14 h at 4°C. Resulting gradients were fractionated into 0.5 ml fractions collected from the bottom of the tube. mtDNA was extracted by phenol–chloroform extraction and resolved on 1% agarose gels.

### Immunoprecipitation of ICT1-FLAG and MRPS27-FLAG

Mitochondria isolated from HEK293T cells overexpressing ICT1-FLAG or MRPS27-FLAG were lysed with 50 mM Tris HCl pH 7.4, 150 mM NaCl, 1 mM EDTA, 1% Triton X-100. Immunoprecipitation was performed with anti-FLAG M2 beads following manufacturer's instructions (Sigma). Elution was performed with the 3X FLAG peptide (Sigma).

### Analysis of mitochondrial ribosomes on sucrose gradients

For the analysis of separate large and small mitochondrial ribosomal subunits, whole cells were solubilized in lysis buffer (50 mM Tris pH7.4, 150 mM NaCl, 1 mM EDTA, 1% Triton), supplemented with 1× proteinase inhibitor cocktail without EDTA (Roche) and loaded onto linear 10–30% sucrose gradients (gradient buffer: 50 mM Tris-HCl pH 7.2, 10 mM Mg(OAc)_2_, 80 mM NH_4_Cl, 100 mM KCl) and centrifuged at 100 000 *g* for 2 h 15 min at 4°C. Gradients were collected as 22 × 100 μl fractions from the top of the gradient. Intact mitochondrial ribosomes were fractionated using a protocol adapted (15). Mitochondria were isolated from four 80% confluent 175 cm^2^ flasks using EDTA free buffers. Then, 1 mg lots of mitochondria were lysed in 260 mM sucrose, 100 mM KCl, 20 mM MgCl_2_, 10 mM Tris-HCl (pH 7.5), 1% Triton X-100, EDTA-free complete protease inhibitor (Roche), and 0.08 U/ml RNAsin (Promega) for 20 min on ice. Mitochondrial lysates were cleared by centrifugation (10 000 *g* for 45 min at 4°C) and loaded on a 10–30% linear sucrose gradient containing 100 mM KCl, 20 mM MgCl_2_, 10 mM Tris-HCl (pH 7.5), and EDTA-free complete protease inhibitor (Roche). After centrifugation at 71 000 *g* for 15 h at 4°C, 15 fractions of 750 μl were collected from the top of the gradient using an automated gradient harvester (Brandel) and subjected to western blot analysis.

### Quantitative real-time PCR for estimation of mtDNA copy number and *MPV17L2* mRNA level

Q-PCR was performed on 25 ng lots of total cellular DNA, using portions of the COII and cytochrome *b* genes for mtDNA and APP1 for nuclear DNA. Primers with the following sequences were employed: COXII, forward 5′-CGTCTGAACTATCCTGCCCG-3′, reverse 5′-TGGTAAGGGAGGGATCGTTG-3′, probe 5′-CGCCCTCCCATCCCTACGCATC-3′; Cytb forward 5′-GCCTGCCTGATCCTCCAAAT-3′, reverse 5′-AAGGTAGCGGATGATTCAGCC-3′, probe 5′-CACCAGACGCCTCAACCGCCTT-3′. Probes contained a 5′ FAM fluorophore and a 3′ TAMRA quencher (Sigma Genosys). For the nuclear reference gene, a validated (20×) APP TaqMan Copy Number Assay master mix was used (Applied Biosystems ID Hs00339475_cn) containing primers and probe. For *MPV17L2* messenger RNA (mRNA) abundance estimation, total RNA was extracted using TRIzol reagent (Life Technologies) according to manufacturer's instructions. DNA was removed with the Turbo DNase free kit (Applied Biosystems) and reverse transcription was performed using Omniscript reverse transcriptase (QIAGEN) with random hexamer primers (QIAGEN) to produce single-stranded cDNA template. Primers and probes to MPV17L2 (TaqMan, Life Technologies, Assay ID: Hs00261946_m1, FAM reporter and non-fluorescent quencher) and endogenous control *GAPDH* (Sigma Genosys, Probe: FAM reporter and TAMRA quencher: 5′-[6FAM]ATTTGGTCGTATTGGGCGCCTGGT[TAM]-3′, forward: 5′-GGTGAAGGTCG GAGTCAACG-3′ reverse: 5′-CAGAGTTAAAAGCAGCCCTGGT-3′) were used to estimate *MPV17L2* mRNA level using 100ng sscDNA in each reaction. TaqMan gene expression assay mix (Life Technologies) and default cycling parameters on the Applied Biosystems (ABI) sequence detection system 7700 were used.

### SDS-PAGE, western blotting and immunoblotting detection

Protein samples were prepared in 1× Laemmli loading buffer, heated at 42°C for 45 min and resolved on the indicated PAGE gels (Novex). After electrophoresis resolved proteins were transferred to a polyvinylidene fluoride membrane. After blocking with 5% non-fat dry milk in PBS with 0.1% (v/v) Tween-20 (PBST) membranes were incubated overnight with primary antibodies. Primary antibodies employed for immunoblotting were: rabbit anti-AIF (Apoptosis-inducing factor) (1:4000, Millipore), rabbit anti-C-terminal of ATAD3 (1:50 000, was raised against recombinant protein produced in-house), rabbit anti-N-terminal of ATAD3 (1:30 000, was raised against recombinant protein produced in-house), rabbit anti-C7-orf30 (1:250, Abcam), mouse anti-Core1 (1:2000, Invitrogen), mouse anti-Core2 (1:2000, Invitrogen), mouse anti-Cox1 (1:1000, Mitoscience), mouse anti-Cox2 (1:1000, Mitoscience), rabbit anti-ERAL1 (1:1000, Proteintech), mouse anti-FLAG (1:1000, Sigma), mouse anti-GAPDH (1:20 000, Sigma), rabbit anti-HSP60 (1:10 000, Abcam), rabbit anti-LRPPRC (1:3000, Santa Cruz Biotechnology), rabbit anti-MPV17 (1:500 Proteintech), rabbit anti-MPV17L2 (1:500, Abcam), goat anti-MRPL3 (1:1000, Abcam), rabbit anti-MRPL11 (1:1000, Cell Signaling); rabbit anti-MRPL12 (1:1000, Proteintech), rabbit anti-MRPL13 (1:1000, Proteintech), rabbit anti-MRPL45 (1:1000, Genetex), rabbit anti-MRPL48 (1:1000, Proteintech), rabbit anti-MRPL58 (Ict1, 1:1000, Proteintech), rabbit anti-MRPS17 (1:1000, Proteintech), rabbit anti-MRPS18B (1:1000; PTGLAB); rabbit anti-MRPS22 (1:1000, Proteintech), rabbit anti-MRPS26 (1:1000, Abcam), rabbit anti-MRPS27 (1:1000, Proteintech), mouse anti-MRPS29 (DAP3, 1:1000, Abcam), mouse anti-NDUFA9 (1:1000, Mitoscience), rabbit anti-TFAM (1:50 000 a kind gift of Prof. R. Wiesner), rabbit anti-prohibitin (1:500 BioLegend), rabbit anti-TOM20 (1:20 000, Santa Cruz), rabbit anti-TIM23 (1:2000, Sigma), mouse anti-VDAC1 (1:4000, Abcam). Secondary HRP antibodies were obtained from Promega and used at 1:5000 in 5% milk in 1× PBS/0.1% Tween. Immunoblots were developed using enhanced chemiluminescence (ECL) and ECL Prime (GE Healthcare).

### **[** ^35^S]-methionine in cell labelling of mitochondrial proteins

Mitochondrial translation products in cultured cells were labelled as described previously (16). HeLa cells, transfected with non-targeting siRNAs or siRNAs targeting *MPV17L2*, or mock transfected, were washed twice with methionine/cysteine-free DMEM (Sigma) supplemented with 2 mM l-glutamine, 96 μg/ml cysteine and 5% (v/v) dialyzed FBS followed by 10 min incubation in this media at 37°C. Cytosolic translation was subsequently inhibited by incubating the cells for 20 min with 100 μg/ml emetine dihydrochloride (Sigma). 100 μCi [^35^S]-methionine was added and labelling was performed for 1 h at 37°C, after which cells were washed three times with PBS (Life Technologies) before lysis in 1× PBS, 0.1% *n-*dodecyl-β-d-maltoside (DDM), 1% SDS, 50 units benzonase (Novagen), 1:50 (v/v) Roche protease inhibitor cocktail. Twenty micrograms lots of protein were resolved via SDS-PAGE (Novex) and the radiolabelled proteins were detected by Phosphorimager of the dried gels (Typhoon Molecular Imager FX, GE Healthcare).

### Phylogenic analysis

The MPV17 protein family was assembled via a PSI-Blast search (default parameters), starting with the human MPV17 protein and iterating until convergence. Members of the family were selected to delineate the origin of the four members of the family in mammals (MPV17 MPV17L, MPV17L2 and PMP22) and based on the presence of experimental data (mainly protein locations, obtained from SUBcellular location database for Arabidopsis proteins (SUBA) (http://www.suba.bcs.uwa.edu.au)). Subsequently, a protein sequence alignment was created with ClustalX (17), followed by minor manual modifications. Based on this alignment, the most appropriate model for MPV17 sequence evolution (LG + G + F) and the corresponding tree were selected with ProtTest (18) after which the bootstrap values were obtained with PhyML (19).

### Subfractionation of yeast mitochondrial proteins

Sym1 tagged with a triple HA tag at its C-terminus was expressed in *Saccharomyces cerevisiae*. Mitochondria were isolated as described previously (20). After rabiolabelling of mitochondrial translation products with ^35^S-methionine *in organello*, the mitochondria were lysed with 1% Triton X-100, 50 mM NH_4_Cl, 5 mM MgSO_4_, 1 mM phenylmethylsulfonyl fluoride, 20 mM HEPES, pH 7.4. After a clarifying spin (10 min at 25 000 *g*, 4°C), the lysate was loaded on a linear sucrose gradient (12 ml, 10–34% sucrose, 0.1% Triton X-100, 50 mM NH_4_Cl, 5 mM MgSO_4_ and 20 mM HEPES pH 7.4). The samples were centrifuged in an SW41 rotor (Beckman) at 33 000 rpm for 5.5 h at 4°C. Subsequently, 16 fractions were collected and containing proteins were precipitated with TCA.

### RNA extraction and northern blotting

Total RNA from HEK293T cells was extracted using Trizol (Invitrogen) via chloroform extraction and isopropanol precipitation according to manufacturer's specifications. Five to eight micrograms of total RNA was resolved on 1% agarose gels containing 0.7 M formaldehyde, in 1× MOPS (3-(N-morpholino) propanesulfonic acid) buffer. Resulting gels were imaged under ultraviolet (UV). RNA was transferred onto MagnaProbe nylon membrane (GE) in 5× SSC, 10 mM NaOH and RNA was UV-crosslinked to the membrane. Membranes were probed with radioactively labelled PCR fragments. PCR products were labelled with ^32^P-dCTP (Hartmann Analytic) using DNA Polymerase I Klenow Fragment (New England Biolabs). Forward and reverse primers for probes were as follows 5′-3′: ND1, CATGGCCAACCTCCTACTCCTCATT and GGCAGGAGTAATCAGAGGTGTTCTTG; A6/COII, TATTCCTAGAACCAGGCGACCTGC and TTTCGTTCATTTTGGTTCTCAGGGT TG; CYTB, CCCCCATAAATAGGAGAAGGCTTAGAAG and CCCGATGTGTAGGAAGA GGCAG; 18S rRNA GTTGGTGGAGCGATTTGTCT and GGCCTCACTAAACCATCCAA.

## RESULTS

### MPV17 belongs to a family of evolutionary conserved proteins

**Figure 1.**
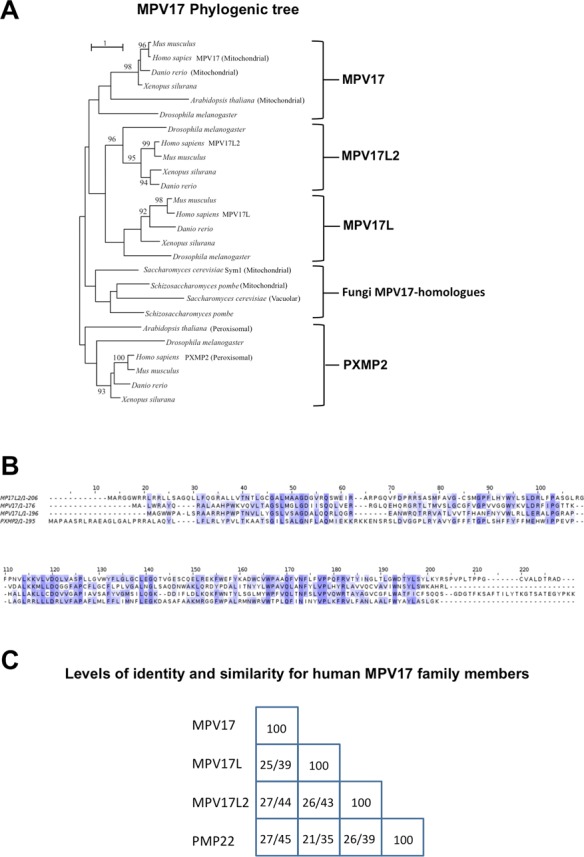
The MPV17 family of proteins. (**A**) Maximum likelihood phylogeny of the MPV17 protein family, with an emphasis on the origin of the human members of the protein family. Experimental localization data for proteins are indicated with the branches. Bootstrap values above 90 out of 100 are shown. The phylogeny indicates an early separation of the MPV17 clade from the PXMP2 clade, and a later, metazoan origin of the MPV17L and MPV17L2 clades. Overall there is a general pattern of lineage specific duplications throughout the eukaryotes, leading, for example, to 10 members of the MPV17 protein family in *Drosophila melanogaster* (not all of which are shown). Protein location within an orthologous group tends to be conserved, but gene duplications often leads to the acquiring of a new location (60), as can be observed for the *PXMP2/MPV17* gene duplication at the origin of the eukaryotes and the gene duplication within the fungi that potentially give rise to a new, vacuolar location of *YOR292C* (61). (**B**) Sequence alignment of the four members of the human MPV17 family. Alignment was based on a ClustalX alignment of the protein family. Colour coding of the level of conservation was based on Jalview (62). (**C**) Levels of sequence identity/similarity among the human MPV17 family members, the ordering of the proteins along the horizontal axis is identical as that along the vertical axis. Pairwise levels of sequence identity/similarity were based on global pairwise sequence alignment, using default parameters in the EMBOSS package (63). MPV17L2 has a slightly higher level of sequence identity and sequence similarity to MPV17 than MPV17L has to MPV17.

To map the origin of the human MPV17 homologues and to resolve the orthology relationships between them and MPV17 homologues from other species, we derived an MPV17 phylogeny (Figure 1A). The comparisons of the MPV17 related proteins from human, from model organisms with protein localization data, and from representative taxa in the metazoa support their classification as a small conserved family. The phylogeny indicates that a gene duplication event before the radiation of the eukaryotes produced a MPV17/L/L2 clade on one hand and a PXMP2 clade on the other hand. Later gene duplication events early in metazoan evolution gave rise to the MPV17, MPV17L and MPV17L2 clades and the phylogeny suggests that MPV17L and MPV17L2 are sister groups of each other. Pairwise comparisons of the three human amino acid sequences show that MPV17L2 has a higher level of sequence similarity and identity to MPV17 than has MPV17L (Figure 1B and C), which is reflected in the relatively long branch lengths in the MPV17L clade compared to the MPV17L2 clade (Figure 1A). Therefore, we began the study of the wider MPV17 family with MPV17L2, with the ultimate aim of understanding both its function and that of other family members, especially MPV17, deficiencies of which cause mtDNA maintenance disorders.

### Human MPV17L2 is an integral mitochondrial membrane protein

**Figure 2.**
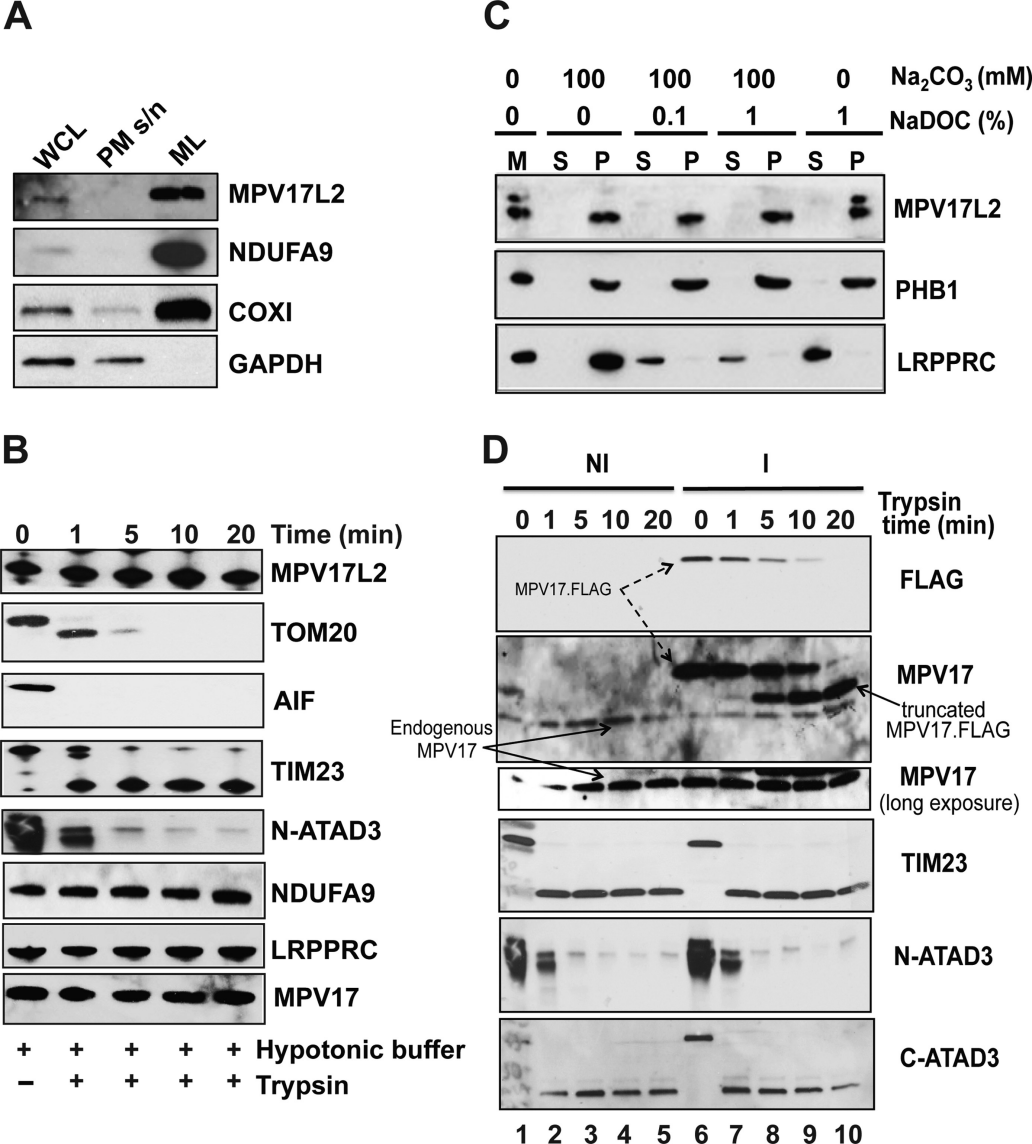
MPV17L2 is an integral membrane protein of the mitochondrial inner membrane. (**A**) Cellular fractionation of HEK293T cells. Proteins from whole cell lysate (WCL, 20 μg), post-mitochondrial supernatant (PM s/n 20 μg) and mitochondrial lysate (ML: 20 μg) were analysed by immunoblotting with the indicated antibodies. Mitochondrial markers were NDUFA9 (nuclear encoded subunit of Complex 1) and COX1 (mtDNA encoded subunit of cytochrome *c* oxidase), whereas GAPDH antibody was used as an indicator of cytosolic protein content. MPV17L2 was found to be enriched in the mitochondrial lysate. (**B**) Time course of limited proteolysis of HEK293T parental mitochondria in hypotonic buffer. Proteins were resolved by 4–12% gradient, or 12 or 16% linear SDS-PAGE according to the mass of the target protein and analysed by immunoblotting, using antibodies against the OMM (TOM20), the IMS (AIF), the IMM (TIM23, the N-terminal and C-terminal domains of ATAD3, NDUFA9, MPV17) and the matrix (LRPPRC) proteins (see text for details). (**C**) Analysis of the solubility MPV17L2. Isolated mitochondria were extracted with sodium carbonate at pH 11.5, with 0, 0.1 or 1% sodium deoxycholate (NaDOC). In all conditions MPV17L2 and PHB1 remained in the pellet (P), whereas LRPPRC was released to the supernatant (S) after sodium carbonate extraction; M, untreated mitochondria. (**D**) As (B), except that mitochondria were isolated from HEK293T cells not-induced (NI, lanes 1–5) or induced (I, lanes 6–10), to express recombinant MPV17-FLAG-STREPII. Mitoplasts were analysed by 12 or 16% SDS-PAGE and immunoblotted with MPV17 (black arrow) and the FLAG epitope (dashed arrow) antibodies. The antibodies against TIM23, the N- and C-termini of ATAD3 were used as IMS and IMM controls, respectively. The lowest panel is a long exposure of the immunoblot for endogenous MPV17.

To determine whether or not mammalian MPV17L2 is a mitochondrial protein, HEK293T cells were fractionated and analysed by immunoblotting, using an antibody to MPV17L2. A protein of the expected size, enriched in mitochondria, cross-reacted with the MPV17L2 antibody, suggesting that MPV17L2 is targeted to the mitochondria. No signal was detected in the post-mitochondrial supernatant (Figure 2A). To establish the location of MPV17L2 within the organelle, mitochondria from HEK293T cells were subjected to hypotonic shock and mild proteolysis treatment. This procedure degrades contaminants, outer mitochondrial membrane (OMM), and intermembrane space (IMS) proteins, as well as any part of an inner mitochondrial membrane (IMM) protein that projects into the IMS. Its effectiveness is demonstrated here by the digestion of TOM20 and AIF, markers of OMM and IMS respectively, the IMS portion of the IMM protein TIM23 (21) and the amino terminal portion of most ATAD3A molecules (14) (Figure 2B). In contrast, the mitochondrial inner membrane remained intact, as evidenced by the preservation of the IMM protein NDUFA9, and a mitochondrial matrix protein LRPPRC (Figure 2B). MPV17L2 is fully resistant to the treatment, indicating it resides in the IMM, or the mitochondrial matrix. To distinguish between these last two possibilities, trypsin-treated mitochondria from HEK293T cells were subjected to alkaline stripping and deoxycholate treatment to lyse the mitochondria and release matrix proteins and peripheral membrane proteins into solution (22,23). MPV17L2 was detected in the insoluble pellet fraction (P), with little or none in the supernatant (S), under all conditions tested (Figure 2C). The mRNA binding protein LRPPRC (24) was released into the supernatant by the alkaline treatment, whereas the established IMM protein prohibitin 1 (PHB1) (25) remained in the pellet (Figure 2C). Together the results indicate that MPV17L2 is firmly embedded in the IMM. The related protein MPV17 is also resistant to alkaline and deoxycholate stripping (3), and like MPV17L2 it is resistant to limited proteolysis after rupturing of the OMM (Figure 2B). In light of these data, it can be concluded that no part (tail or loop) of MPV17 or MPV17L2 projects into the IMS sufficiently to permit trypsin degradation, which is compatible with both the amino and carboxyl terminal tails (of both proteins) facing the matrix. Nevertheless, experimental data from a tagged form of Sym1, the yeast mitochondrial orthologue of MPV17, suggest that the C-terminus of the protein ‘faces’ the IMS (26,27). Because we had available a carboxy terminal tagged form of MPV17, additional experiments were performed to clarify the orientation of this protein. Hypotonic shock and trypsin treatment of mitochondria isolated from cells expressing the recombinant protein resulted in digestion of part, but not all, of the FLAG and StrepII tags, as the size of the product was larger than endogenous MPV17 (Figure 2D). Thus, as with Sym1: at least the carboxyl terminus of recombinant MPV17 faces the IMS. However, recombinant MPV17, and by extrapolation the endogenous protein, is embedded in the IMM or otherwise arranged to protect it from trypsin digestion (Figure 2D). Clearly this finding has implications for the study of IMM proteins generally: the inability to digest one or other part of an IMM protein cannot be taken as evidence that it is matrix facing.

### Decreased *MPV17L2* expression causes mitochondrial swelling and nucleoid aggregation

**Figure 3.**
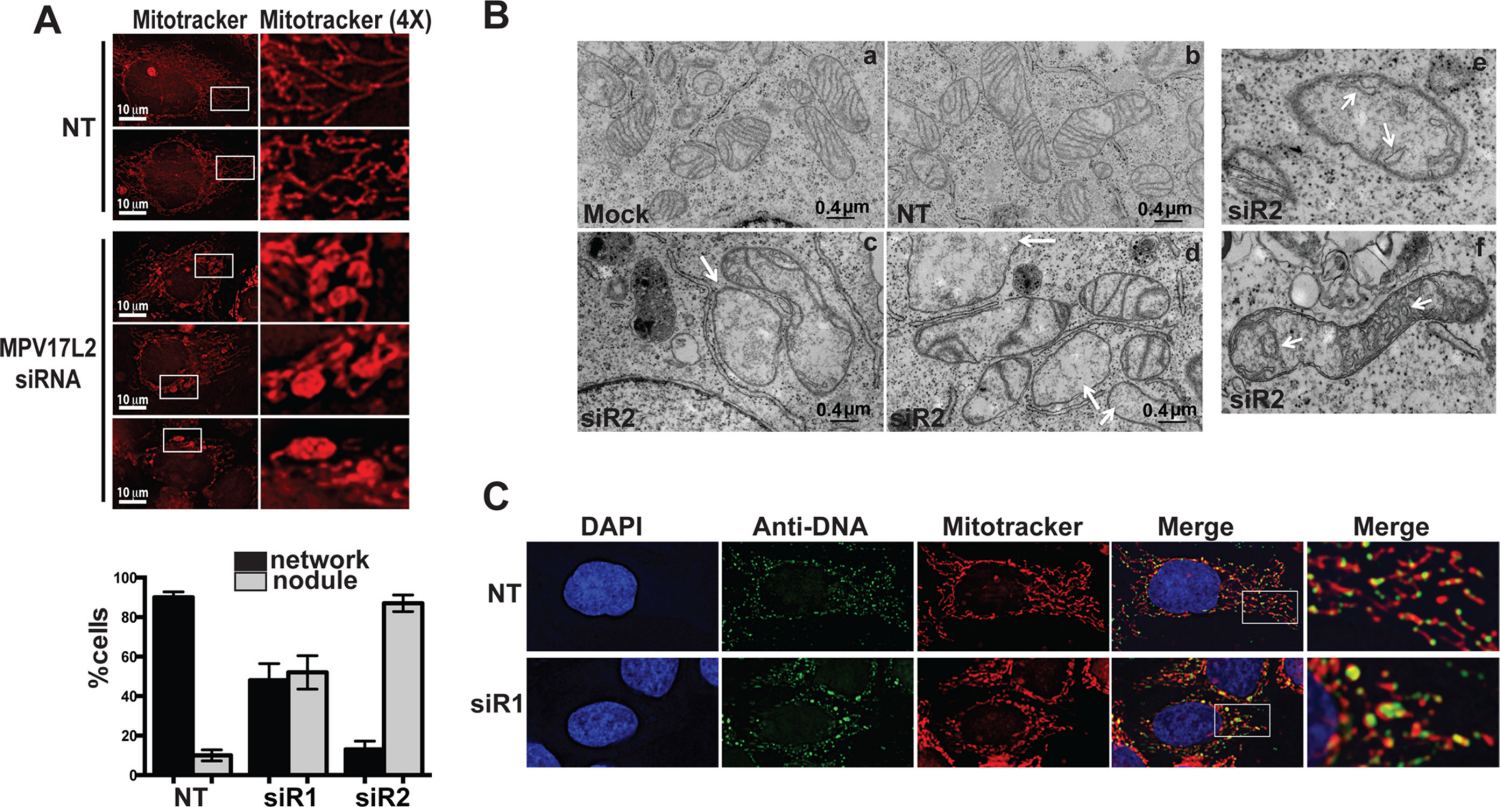
*MPV17L2* gene silencing affects mitochondrial and nucleoid morphology. After two rounds of transfection with one of three siRNAs targeting *MPV17L2* or a non-target (NT) dsRNA, the mitochondria of HeLa cells were stained with MitoTracker™ Red (**A**) (see ‘Materials and Methods’ section for details). MitoTracker™ dense areas (nodules) were scored in 100 control (NT) and 100 *MPV17L2* siRNA-treated cells (*n* = 2 experiments, 50 cells per experiment). The same phenotype of increased numbers of distended mitochondria was observed on five independent experiments but not quantified. (**B**) Mitochondria of HeLa cells treated with no RNA (Mock) (a), or non-targeting dsRNA (NT) (b), or dsRNAs targeting *MPV17L2* (siR2) (c–d) were visualized by electron microscopy. Arrowheads indicate mitochondria devoid of cristae (c and d), or with distended cristae (e and f). The scale bars in panel a–d are 0.4 μm. The images in panels e and f were enlarged to show the altered cristae structure in more detail (1.5×). (**C**) Confocal microscopy of HeLa cells transfected with non-targeting (NT) or *MPV17L2*-specific dsRNA (siR1). Mitochondria were stained with MitoTracker™ Red, the DNA in the cytoplasm was detected by immunocytochemistry (green) and nuclear DNA was detected by DAPI staining (blue).

An idea of the contribution of a protein to mitochondrial structure and function can often be gleaned by examining the effect of gene silencing on the organelles. After downregulation of *MPV17L2* expression in HeLa cells (Supplementary Figure S1), with any of three specific siRNAs, there were many prominent protrusions or nodules in the mitochondrial network, unlike cells transfected with non-target oligonucleotides (Figure 3A). To investigate the ultrastructure of the abnormal mitochondria the HeLa cells were analysed by electron microscopy. After *MPV17L2* gene silencing many mitochondria were enlarged and the cristae were sparse or completely absent, in contrast to the cells transfected with a non-target dsRNA, (Figure 3B). In some mitochondria of cells depleted of MPV17L2 the cristae were swollen, which might well precede cristae loss (Figure 3B). Detection of mtDNA by immunocytochemistry indicated that the enlarged nodules in the mitochondria coincided with high concentrations of DNA (Figure 3C). Hence mtDNA distribution or segregation appears to be impaired in the absence of MPV17L2. Although many nucleoids were larger than normal, similar to *TFAM* gene silencing (28), decreased *MPV17L2* expression did not lead to mtDNA depletion within the timeframe of the experiment (Supplementary Figure S2A), unlike TFAM repression (28). Instead, there was ∼70% increase in mtDNA copy number in response to *MPV17L2* gene silencing and no marked change in mitochondrial transcript levels (Supplementary Figure S2B).

### MPV17L2 co-fractionates with mitochondrial nucleoids on iodixanol gradients and is not detectable in cells devoid of mtDNA

**Figure 4.**
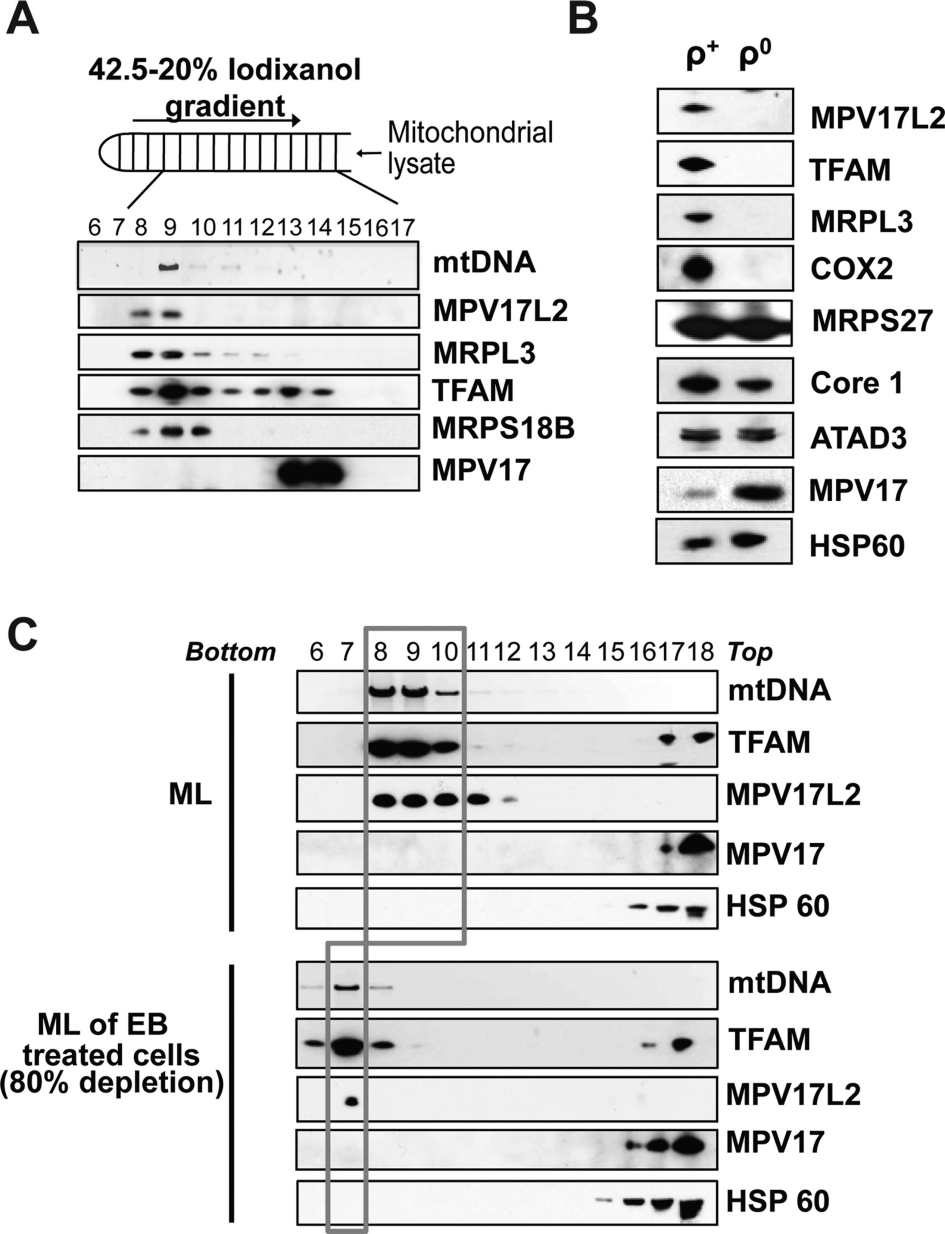
MPV17L2 distribution overlaps with mtDNA on iodixanol gradients, whilst in cells lacking mtDNA MPV17L2 is not detectable. (**A**) Mitochondrial 1000 g_max_ supernatants from HEK293T cells were fractionated on 20–42.5% iodixanol gradients, the DNA content of each fraction was determined by ethidium bromide staining after agarose gel electrophoresis. Proteins were separated on 4–12% gradient, or 12 or 16% linear SDS-PAGE, proteins were detected by immunoblotting with the indicated antibodies (see text for details). (**B**) Immunoblots of total cellular protein from HEK293T cells with (ρ^+^) and without (ρ^0^) mtDNA. ATAD3, ATPase family AAA domain-containing protein 3; COX2, Cytochrome *c* Oxidase subunit 2; Core1, nuclear subunit Complex III; HSP60, Heat Shock Protein 60; MRPL3, MPV17, homologous protein to MPV17L2; Mitochondrial Ribosomal Large Subunit Protein 3; MRLS18B, Mitochondrial Ribosomal Small Subunit Protein 18B; MRLS27, Mitochondrial Ribosomal Small Subunit Protein 27; TFAM, mitochondrial Transcription Factor A. (**C**) HEK293T cells were treated with or without 100 ng/ml ethidium bromide (EB) for 72 h. Mitochondrial lysates (ML) (see ‘Materials and Methods’ section) were fractionated on 20–42.5% iodixanol gradients. DNA or protein was extracted after fraction collection and analysed by Southern hybridization (mtDNA) and immunoblotted with the indicated antibodies.

To determine if the mtDNA phenotypes associated with MPV17L2 depletion (Figure 3) reflected a direct interaction between the two mitochondrial nucleoprotein complexes were resolved on iodixanol gradients (IG) and analysed by immunoblotting. MPV17L2 co-fractionated with mitochondrial nucleoids (Figure 4A), and in cells lacking mtDNA MPV17L2 was undetectable (Figure 4B). Furthermore, the abundance of MPV17L2 decreased in line with mtDNA copy number, when mtDNA depletion was induced by ethidium bromide (EB) treatment of HEK293T cells for 72 h (Figure 4C); in these circumstances the mtDNA resolves lower on the iodixanol gradient, and this was also true of MPV17L2. The dependence of MPV17L2 on the presence of mtDNA suggests it is involved in DNA maintenance or expression. In contrast to MPV17L2, the bulk of the MPV17 protein resolves much higher on the IG than mtDNA (Figure 4A), and it persists in cells lacking mtDNA (Figure 4B).

### MPV17L2 co-sediments with the large subunit of the mitochondrial ribosome and the monosome

**Figure 5.**
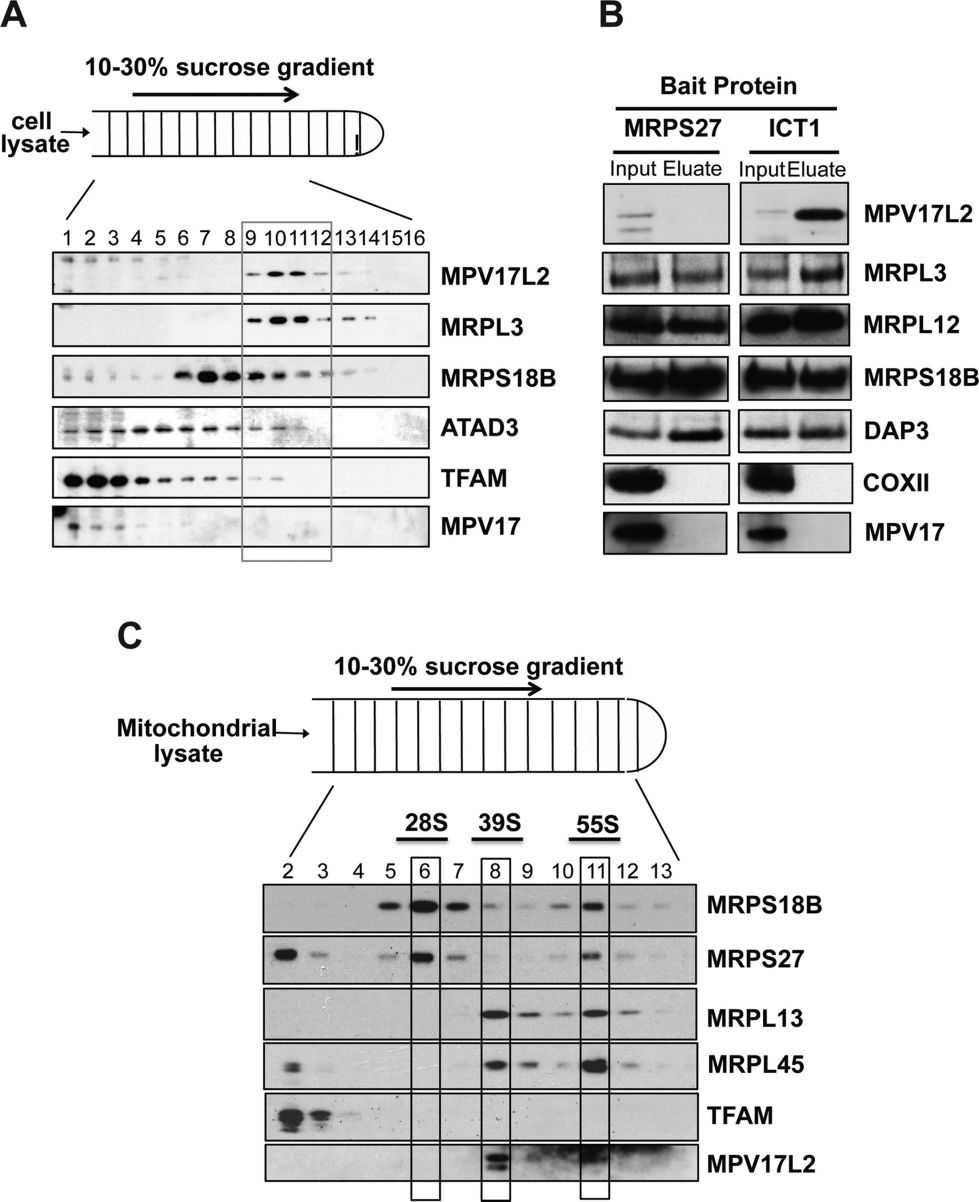
MPV17L2 interacts with the large subunit of the mitochondrial ribosome and the monosome. (**A**) HEK293T cells were lysed and their contents fractionated on 10–30% sucrose gradients. The distribution of representative mitochondrial nucleoid and ribosomal proteins was determined by immunoblotting (see ‘Materials and Methods’ section and text for details). (**B**) HEK293T cells expressing MRPS27-FLAG (a component of mt-SSU) or ICT1-FLAG (a component of mtLSU) were lysed and subjected to immunoprecipitation with FLAG-antibody. The lysates (‘input’) and eluates were analysed by immunoblotting with the indicated antibodies. (**C**) HeLa cell mitochondria lysed in EDTA-free buffer were fractionated on 10–30% sucrose gradients. The migration of the monosome and the mtLSU and mtSSU were inferred from the distribution of individual ribosome components detected by immunoblotting using the indicated antibodies.

Mitochondrial ribosomes and associated proteins coincide or overlap with mitochondrial nucleoids on iodixanol gradients (13), and the mitochondrial ribosomal protein MRPL3 shows the same dependence on, and association with, mtDNA as MPV17L2 (Figure 4A, 4B). Hence, the properties of MPV17L2 described above were compatible with it interacting primarily with the mitochondrial ribosome. Therefore, sucrose gradients (SG) were used to separate mitochondrial ribosomes from the nucleoids and fractions of the gradients were immunoblotted with antibodies to MRPL3 and MRPS18B to determine the distribution of the large mitochondrial ribosomal subunit (mtLSU) and the small mitochondrial ribosomal subunit (mtSSU), respectively. Based on these markers MPV17L2 co-sediments with the mtLSU and not the mtSSU (Figure 5A).

As a further test of the association of MPV17L2 with the mitochondrial ribosome, immunoprecipitation was performed using HEK293T cells expressing ICT1-FLAG, a component of the mtLSU (29), or MRPS27-FLAG, a component of the mtSSU (30). Endogenous MPV17L2 was greatly enriched in immunoprecipitations using ICT1-FLAG (Figure 5B), whereas it was undetectable when MRPS27-FLAG was used as bait. Hence, the immunoprecipitation experiments substantiate the sucrose gradient results indicating MPV17L2 associates with the mtLSU. They also corroborated the sucrose-gradient results with respect to MPV17, the related family protein: MPV17 did not co-fractionate with either the large or the small mitochondrial ribosomal subunit (Figure 5A) and nor was it enriched in the immunoprecipitations of ICT1-FLAG or MRPS27-FLAG (Figure 5B).

Having learnt that MPV17L2, and not MPV17, interacts with the mitochondrial ribosome, this distinguishing feature was explored in budding yeasts which have a single mitochondrial MPV17 homologue, Sym1. To this end, a sequence cassette coding for an HA tag was integrated into the chromosome downstream of the Sym1 locus resulting in the expression of an HA-tagged Sym1 protein. Fractionation of mitochondrial proteins on sucrose gradients revealed that Sym1 was not associated with the mitochondrial ribosomes (Supplementary Figure S3). Thus, it is unlikely that Sym1 incorporates all the functions of the two human proteins MPV17 and MPV17L2, and the latter probably gained a new function after the gene duplication event.

The experiments described above were carried out in buffer containing EDTA and without addition of high concentration of magnesium. Therefore, mitochondria from HeLa cells were isolated in EDTA-free buffer, disrupted in the presence of 20 mM magnesium and sedimented on sucrose gradients (see ‘Materials and Methods’ section) to determine if MPV17L2 associates with the 55S mitochondrial ribosome. Co-fractionation of a proportion of MRPL13, MRPS27 and MRPS18 (Figure 5C, fraction 11) confirmed the preservation and location of the monosomes on the gradient, and a similar proportion of the MPV17L2 protein was also present in fraction 11, suggesting that it physically interacts with fully assembled mitochondrial ribosomes.

### *MPV17L2* gene silencing inhibits mitochondrial translation

**Figure 6.**
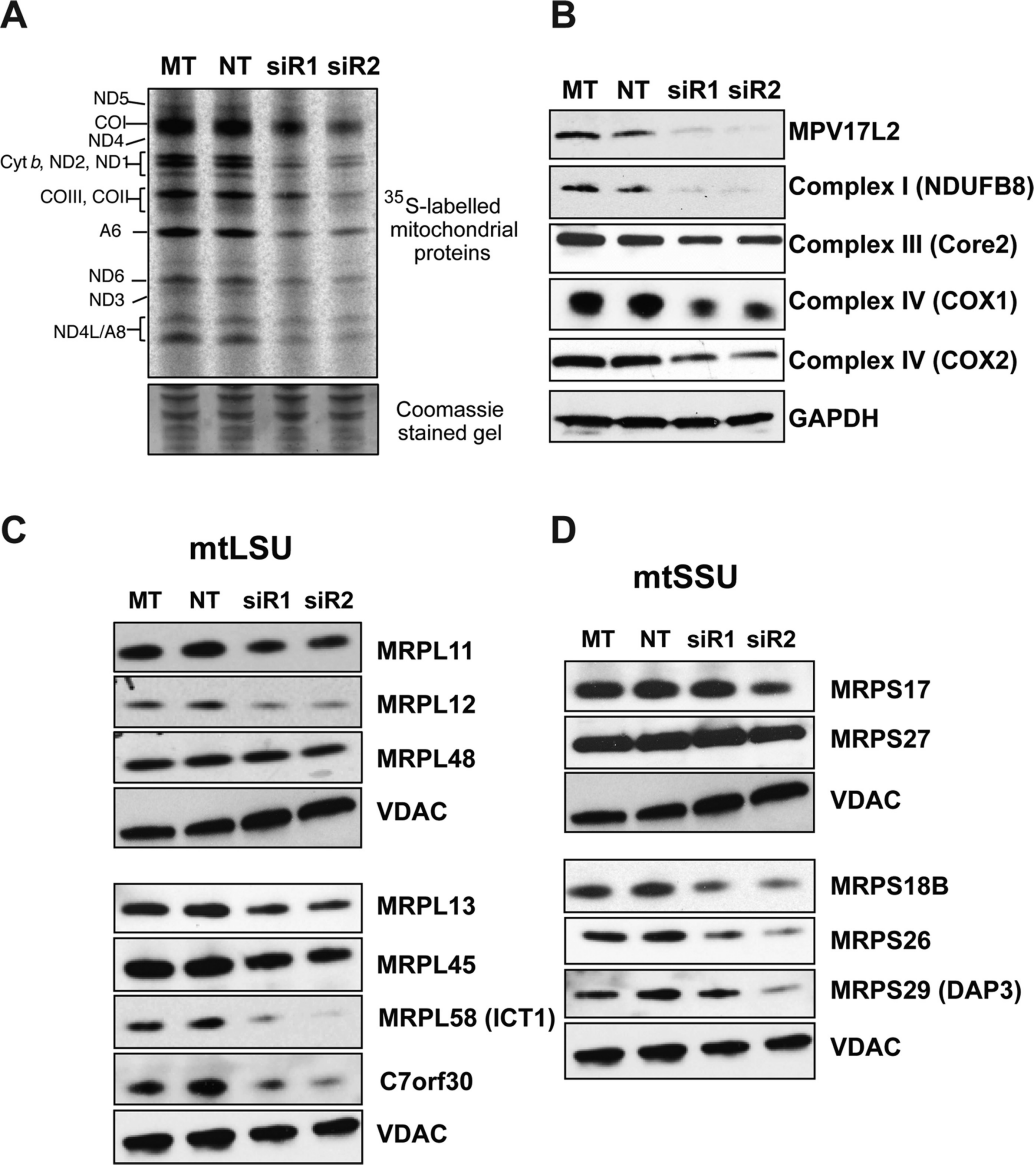
*MPV17L2* gene silencing inhibits mitochondrial protein synthesis and decreases the abundance of OXPHOS and mitochondrial ribosomal proteins. Expression of *MPV17L2* was repressed by RNA interference in HeLa cells (see ‘Materials and Methods’ section) and the effects on mitochondrial translation (**A**), steady state levels of OXPHOS subunits and MPV17L2 (**B**), and MRPs and C7orf30 (**C**), and (**D**) determined. (A) mitochondrial protein synthesis was assayed by incubation of the cells in ^35^S-methionine, after emetine treatment to block cytosolic protein synthesis. Proteins were separated by 12% PAGE and radiolabelled proteins detected by PhosphorImaging. Equal loading of the gels was confirmed by Coomassie blue staining. (B) Protein levels of MPV17L2 and subunits of respiratory chain complexes were analysed by immunoblotting in control cells transfected with no RNA (MT), a random dsRNA (NT) or one of two siRNAs specific for *MPV17L2* (siR1 and siR2). GAPDH was used as a loading control. (C) Steady-state levels of C7orf30 and mtLSU proteins, and (D) mtSSU components. Mitochondria lysates of HeLa cells, treated as in (A) and (B), were separated by SDS-PAGE and immunobloted for the indicated MRPs and VDAC1 as loading control.

Because the sucrose gradient and immunoprecipitation analyses suggested a physical interaction between MPV17L2 and mitoribosomes, we next sought to determine if MPV17L2 contributes to mitochondrial protein synthesis. Transient RNA interference with each one of the three dsRNAs targeting *MPV17L2*, or one of the two random dsRNA sequences, were performed, prior to an assessment of mitochondrial translation capacity in living cells (see ‘Materials and Methods’ section). The synthesis of nascent mitochondrial proteins was markedly reduced in cells transfected with siRNAs targeting *MPV17L2*, compared to controls (Figure 6A and Supplementary Figure S1B). Furthermore, *MPV17L2* gene silencing produced marked decreases in the levels of both nuclear and mitochondrially encoded components of the OXPHOS system (Figure 6B).

### MPV17L2 gene silencing destabilises the mitochondrial ribosome

**Figure 7.**
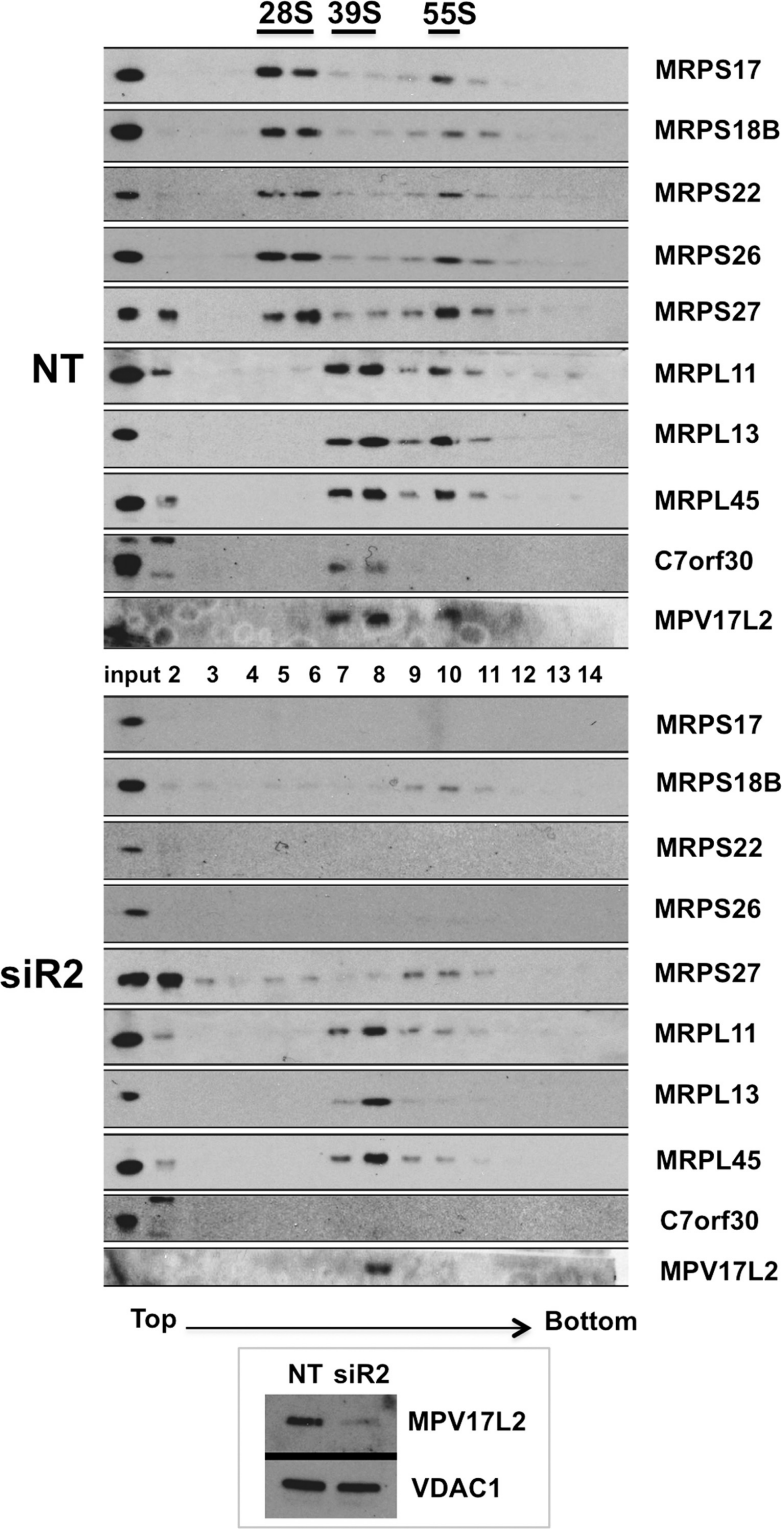
*MPV17L2* gene silencing impairs mitochondrial ribosome assembly. Mitochondrial lysates from HeLa cells treated with a random dsRNA (NT) or a specific siRNA for *MPV17L2* (siR2) were separated on a 10–30% isokinetic sucrose gradient and fractions analysed by immunoblotting with antibodies to MPV17L2, mtLSU (MRPL11, MRPL13, MRPL45), mtSSU (MRPS17, MRPS18B, MRPS22, MRPS26, MRPS27, MRPS29) and C7orf30. Inset: protein levels of MPV17L2 and the loading control VDAC1 in mitochndrial lysates of control and MPV17L2 siRNA treated cells.

We next determined the effect of gene silencing of *MPV17L2* on the steady-state level of ribosomal proteins in HeLa cell mitochondria. Five of six mtLSU components screened were depleted in response to *MPV17L2* RNAi, as was the ribosomal assembly factor C7orf30 (31–33) that associates with the mtLSU (Figure 6C). Less expectedly, several components of the mtSSU were also adversely affected by *MPV17L2* knockdown (Figure 6D). The depletion of the mtSSU components was more striking when the samples were fractionated on sucrose gradients under conditions that preserve the monosome (Figure 7); several mtSSU proteins were at the limits of detection (MRPS17, MRPS22, MRPS29). More informatively, much of the MRPS27, whose steady-state level was not markedly decreased by *MPV17L2* knockdown (Figure 6D), was relocated to the top of the gradient, far above the mtSSU or the monosome (Figure 7). The decreases in the component parts of the mitochondrial ribosome (Figure 6C and D) were mirrored by the amount of monosome, which was markedly decreased as a result of *MPV17L2* silencing (Figure 7). However, there was a residual apparently fully assembled population of mtLSU, based on its sedimentation properties (Figure 7), which contained the remaining MPV17L2. This suggests that there were no mtLSUs lacking MPV17L2, but the residual protein was insufficient to support monosome formation, at least at the normal rate.

**Figure 8.**
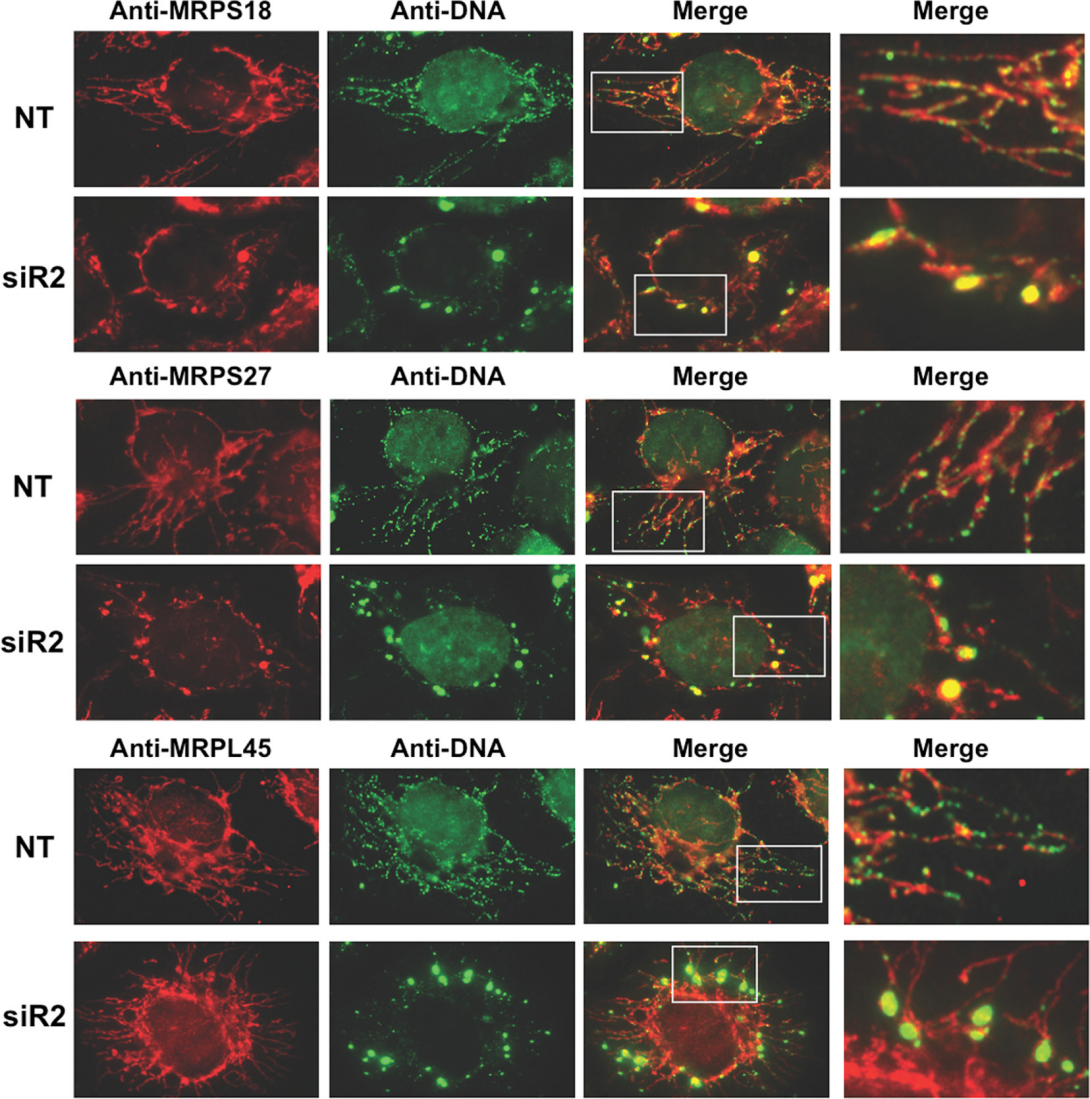
*MPV17L2* gene silencing causes condensation of mtSSU proteins in foci that frequently coincide or overlap with enlarged mitochondrial nucleoids. HeLa cells treated with a random dsRNA (NT) or a specific siRNA for *MPV17L2* (siR2) were analysed by confocal microscopy with antibodies to MRPS18B, MRPS27 and MRPS45 (red), the DNA was detected by anti-DNA (green) antibody. Co-localization of the ribosomal-specific red signal and DNA-specific green signal appears yellow in the merged images.

Because C4orf14 (NOA1) is involved in mtSSU biogenesis and it interacts with mitochondrial nucleoids, it has been proposed that the small subunit is assembled at the mitochondrial nucleoid (34). Furthermore, dissociation of the mtSSU from the nucleoid is presumed to follow its association with the mtLSU (34). The destabilization of the mtSSU in response to depletion of the mtLSU-associated protein MPV17L2 (Figures 5 and 7), suggests that MPV17L2 might mediate unification of the small and large subunits of the mitochondrial ribosome. If true, a dearth of MPV17L2 would prolong the association of mtSSU with mitochondrial nucleoids. In this scenario, the observed decreases in mtSSU (Figures 6D and 7) reflect disassembly or blocked assembly of the 28S subunit to limit the detrimental consequences of the extended contact between nucleoids and mtSSUs. To test this hypothesis we analysed the distribution of the mtLSU, mtSSU and mtDNA in cells treated with *MPV17L2* and non-target siRNAs. Immunocytochemistry of MRPL45, MRPS27 and MPRS18 indicated that both the ribosomal large and small subunits were distributed widely in the mitochondria of control cells. After *MPV17L2* knockdown the distribution of the residual mtLSU (MRPL45) was unaltered, whereas the remaining mtSSU (MRPS27 and MPRS18) was concentrated in a small number of large foci (Figure 8). The effect on the mtSSU was strikingly similar to the effect on the mtDNA, and there was substantial colocalization and juxtaposition of DNA and mtSSU foci (Figure 8). As elaborated in the 'Discussion' section, these findings suggest the mtSSU is assembled at the nucleoid and prolonging the interaction causes mtDNA aggregation.

## DISCUSSION

The current study identifies MPV17L2 as an inner mitochondrial membrane protein with a key role in mitochondrial protein synthesis, as it is required for the assembly and stability of the mitochondrial ribosome. The dependence of MPV17L2 on mtDNA (Figure 4) and its association with the mitoribosome distinguish it from MPV17, and suggest it has evolved a new function after a gene duplication event. Hence, MPV17 and MPV17L2 are paralogues. Both are nuclear genes that influence mtDNA metabolism, MPV17 contributes to mtDNA maintenance (3), whereas MPV17L2 has a role in mitochondrial protein synthesis (this report). Notwithstanding these differences, the immunocytochemistry data indicate that there is a major disturbance of mtDNA organization when MPV17L2 is scarce (Figures 3B and 8), which in view of MPV17L2's ribosomal association suggests perturbed ribosome-nucleoid interactions (13,34,35).

Numerous features of the mitochondrial translation system suggest it has a prokaryotic origin. For instance, many of the proteins linked to mitochondrial ribosomal biogenesis and assembly have clear bacterial homologs and orthologs (13,31,32,34,36–38). In contrast, MPV17L2 does not appear to have any bacterial counterpart linked to protein synthesis. Nevertheless, the protein is concentrated in SG fractions containing the mtLSU or the monosome, with no appreciable free pool of MPV17L2, and there is no contamination of this region of the SG with its paralogue MPV17 (Figure 5A). The ICT1 immunoprecipitation experiments were equally clear, with a substantial enrichment of MPV17L2, and no detectable MPV17, or a subunit of the highly abundant cytochrome *c* oxidase (Figure 5B). Therefore, the function of MPV17L2 can with some confidence be assigned to the workings of the mitochondrial ribosome, and this is corroborated by disruption to the mitochondrial ribosome when its expression is low (Figure 7), and impairment of mitochondrial translation (Figure 6A and Supplementary Figure S1B).

The association of MPV17L2 with the mtLSU on SGs and its immunoprecipitation with ICT1 are features shared with C7orf30 (31,32), a protein which has been proposed to participate in the assembly of the mtLSU (33). Moreover, *MPV17L2* silencing induced a marked depletion of C7orf30 (Figure 6C), and the effects of *C7orf30* gene silencing display considerable overlap with those reported here for MPV17L2. MRPL58 (ICT1) was one of the most severely affected subunits of the mtLSU both in the case of *C7orf30* knockdown (see Figure 4 of (33)) and *MPV17L2* knockdown (Figure 6C). The mtSSU was also affected by both *C7orf30* and *MPV17L2* gene-silencing (Figures 6C, D and 7; and Figure 4 of (33); and Figure 5F of (31). Therefore, we infer that MPV17L2 cooperates with C7orf30 to assemble the mitochondrial ribosome. Furthermore, the adverse effects on the mtSSU of MPV17L2 depletion can be explained by the prolonged association of the mtSSU with the nucleoid. This interpretation is predicated on the idea that the mtSSU is assembled at the mitochondrial nucleoid (34) and that the completion of monosome assembly is a required step for the separation of the mtSSU and the nucleoid. Hence, decreases in the population of mtLSU will extend the time that the mtSSU and the nucleoid remain bound together. Prolonged coupling could well interfere with other aspects of nucleoid function and maintenance, and so the failure to form the monosome can explain the impairment of nucleoid distribution (Figures 3C and 8). Another hypothesis stems from the recent suggestion that both the small and the large ribosomal subunits are in the initial stages assembled at the nucleoid (39); thus if MPV17L2 was involved in incipient mtLSU assembly, its absence might perturb assembly of both ribosomal subunits. However, what argues against this idea is that MRPL45, one of the reported ‘nucleoid-enriched mtLSU components’ identified by Bogenhagen and colleagues (39), was distributed normally in the absence of MPV17L2 (Figure 8). Alternatively, mtSSU depletion owing to a problem relating to the mtLSU might reflect a counting mechanism for the two subunits, whereby a decrease in one invariably leads to downregulation of the other. If true, the counting mechanism over-reacts, as MPV17L2 knockdown depletes the mtSSU to a greater extent than the mtLSU (Figure 7); and this last hypothesis cannot explain the nucleoid aggregation caused by MPV17L2 depletion.

Mitochondrial translation products are highly hydrophobic and are inserted into the inner membrane as they are synthesized (40,41). In yeast, the machinery that couples translation to membrane insertion includes Oxa1, Mba1 and Mdm38 (LETM1), and these proteins physically connect ribosomes to the inner membrane (42–47). In bovine mitochondrial ribosomes, a large additional protein mass has been proposed close to the polypeptide exit tunnel (PET) (48). More recently, a structural component, MRPL45, has been identified at the PET and it has been proposed to anchor the mtLSU to the inner membrane (49). The tight association of MPV17L2 with the mtLSU and the fact that it is firmly embedded in the IMM make MPV17L2 a good candidate for a role at the PET. In particular, it might interact with the nascent polypeptides to facilitate their exit from the ribosome and their insertion into the OXPHOS complexes of the mitochondrial inner membrane. Any interruption to this process could cause ribosome stalling and consequently severely impair mitochondrial translation, as observed when *MPV17L2* is silenced (Figure 6A and Supplementary Figure S1B). The cristae alterations observed in mitochondria of *MPV17L2* knockdown (Figure 3B) are similar to those observed for another protein linked to the mtLSU, CRIF1 (50). Hence, this might be a general consequence of defects in the apparatus of mitochondrial protein synthesis, and it will be of interest to learn whether this is true also of C7orf30, and if it extends to mtSSU-related proteins, such as C4orf14 (34) and ERAL1 (36,38).

The location of MPV17L2 may also serve to stabilize or position the mtLSU and the monosome by linking them to the inner mitochondrial membrane. MPV17L2 may be assisted in this by LETM1 (mdm38), with which it shares a number of features. Both are inner membrane proteins that are associated with mitochondrial ribosomes (43,46,51) and the morphological abnormalities observed in *MPV17L2* downregulation are similar to LETM1 deficient mitochondria (52,53). These similarities strengthen the view that MPV17L2 plays a role in the interlinked processes of translation and assembly of OXPHOS complexes (40,41). LETM1 has another role that might be pertinent to the function of MPV17L2, and that of the wider MPV17 family. LETM1 is proposed to act as K^+^/H^+^ exchanger or calcium proton antiporter (54–56). One member of the MPV17 family, PXMP2 has been proposed to form a channel *in vitro* (10) and Sym1 displayed channel activity in another study (26). Hence, the MPV17 family members might all act as channels with different substrate specificities. If true, a major challenge for the future is to identify the substrate(s) of each family member. There could be some redundancy between MPV17 and MPV17L2, as the expression of MPV17 increased in ρ^0^ cells (Figure 4B), which might compensate to some degree for the loss of MPV17L2. In any case, the increased expression of MPV17 in human cells lacking mtDNA, recapitulates the behaviour of its homologue in yeast (57), thereby indicating that the protein has a function beyond DNA maintenance in human as well as in yeast.

Finally, although there was no decrease in mtDNA copy number after 6 days of MPV17L2 gene silencing (Supplementary Figure S2A), defective mtDNA segregation leading to mtDNA depletion is a likely long-term consequence of nucleoid aggregation. Moreover, this is predicted to be true of any mutant protein that inhibits the uncoupling of the mtSSU from the nucleoid. Hence, mitochondrial ribosomal biogenesis defects might underpin quite a number of the, as yet, unexplained cases of mtDNA depletion syndrome, or provoke neurodegeneration (58). Furthermore, in light of our new findings, translation, rather than transcription (59), could be the key to the age-related accumulation of deleterious mtDNA variants.

## SUPPLEMENTARY DATA

Supplementary Data are available at NAR Online.

SUPPLEMENTARY DATA
