# Supplementary material for: MPV17L2 is required for ribosome assembly in mitochondria
Source: Nucleic Acids Res. 2014 Jun 19;42(13):8500–15. doi: 10.1093/nar/gku513 (PMC4117752; doi:10.1093/nar/gku513)
Supplement: SUPPLEMENTARY DATA [file supp_42_13_8500__index.html]

MPV17L2 is required for ribosome assembly in mitochondria — MPV17L2 is required for ribosome assembly in mitochondria — SUPPLEMENTARY DATA 

# MPV17L2 is required for ribosome assembly in mitochondria

## SUPPLEMENTARY DATA

**Files in this Data Supplement:**

- SUPPLEMENTARY DATA
