# Supplementary material for: MPV17L2 is required for ribosome assembly in mitochondria
Source: Nucleic Acids Res. 2014 Jun 19;42(13):8500–15. doi: 10.1093/nar/gku513 (PMC4117752; doi:10.1093/nar/gku513)
Supplement: SUPPLEMENTARY DATA [file supp_gku513_nar-00094-r-2014-File017.pdf]

SUPPLEMENTARY INFORMATION

For the phylogeny of the MPV17 family the following GenBank identifiers were used (the order of the identifiers is the same as that in the phylogeny, from top to bottom): 6678926 4505241 210147451 156717962 334185594 21356661 21356567 16235468 34147205 52345768 50540200 26024193 190358477 68444055 301617809 161083929 6323280 19115883 398366113 19114608 15233520 21358267 8923892 112421058 52219060 55742326.

Figure S1. RNA interference of *MPV17L2* represses its expression and impairs mitochondrial protein synthesis in HeLa cells. (A) Effective silencing of *MPV17L2* in HeLa cells was confirmed by Q-PCR analysis, using primers specific for *MPV17L2* and *GAPDH* mRNA. Mock transfected (MT), non-targeting dsRNA (NT) or dsRNAs targeting *MPV17L2* (siR1, siR2 or siR3). (**B**) A third siRNA (siR3) targeting *MPV17L2* mRNA impairs mitochondrial protein synthesis. RNA interference was performed on HeLa cells as per figure 6A, except that siR3 was applied in place of siR2. Proteins were separated by 4-12% PAGE and radiolabelled proteins detected by PhosphorImaging. Equal loading of the gels was confirmed by Coomassie blue staining. (**C)** Knockdown of the gene was verified by Q-PCR analysis, using primers specific for *MPV17L2* and *GAPDH* mRNA.

Figure S2. RNA interference of *MPV17L2* increases mtDNA copy number and does not markedly alter the levels of mitochondrial transcripts.
A) Q-PCR estimation of mtDNA copy number in mock transfected (MT), non-targeting dsRNA (NT) or dsRNAs targeting *MPV17L2* (siR1 or siR2). The results were normalized to the MT values,.n = 7 experiments. Data are presented as mean ± SEM. Statistical analysis of differences between the two groups was examined by a two-tailed unpaired Student's *t*-test, ** $p < 0.01$. Silencing of *MPV17L2* was verified at the mRNA level by Q-PCR (see Figure S1) and in some cases additionally at the protein level (e.g. Figure 6B). **B)** RNA was isolated from HeLa cells after two rounds of RNAi to *MPV17L2*. After resolution on 1% agarose gels containing 0.7 M formaldehyde in 1x MOPS buffer, the RNA was transferred to solid nylon support and hybridized to probes corresponding to four mitochondrial transcripts selected at random: ND1, COX2, ATP6 and cytochrome *b*. The positioning of the membrane missed part of the final lane in the case of the membrane probed for cytochrome b and ATP6 genes. Mock transfected (MT), non-targeting dsRNA (NT) or dsRNAs targeting *MPV17L2* (siR1, siR2).

Figure S3. Sym1 does not co-fractionate with the yeast mitochondrial ribosome on sucrose gradients. (**A**) Sym1 tagged at the C-terminus with three haemagglutinin motifs (SYM1-3HA) was expressed in budding yeasts and whole cell proteins immunoblotted with antibody to HA, in parallel with control yeast cell proteins (WT). (**B**) Purified mitochondria expressing SYM1-3HA were fractionated on sucrose gradients, as previously described (61) and the position of the mitochondrial ribosome assigned on the basis of immunoblotting to Mrpl36, which peaks in fractions 10-12, and the distribution of nascent ^{35}S-methionine labelled proteins revealed by autoradiography. Sym1-HA migrated exclusively at the top of the gradient, peaking in fractions 3 and 4. Tom70, a non-ribosomal protein, was found principally in fractions 1 and 2. Thus, Sym1 is not associated with mitochondrial ribosomes under the mild lysis conditions used here (50 mM NH_4Cl). Hence, it behaves like MPV17 and not MPV17L2 (see Figure 5A).

MPV17L2 siRNA

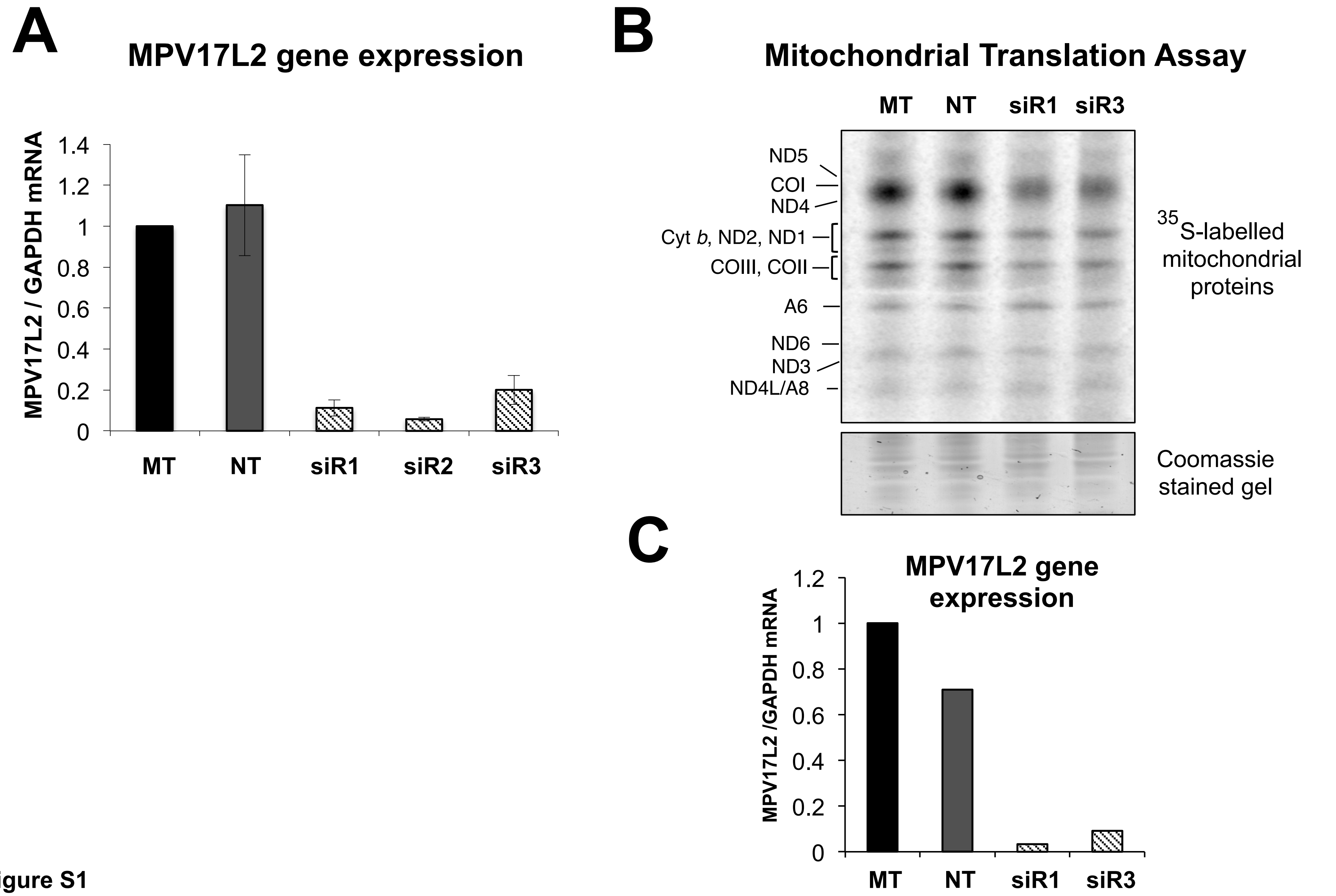

Figure S1

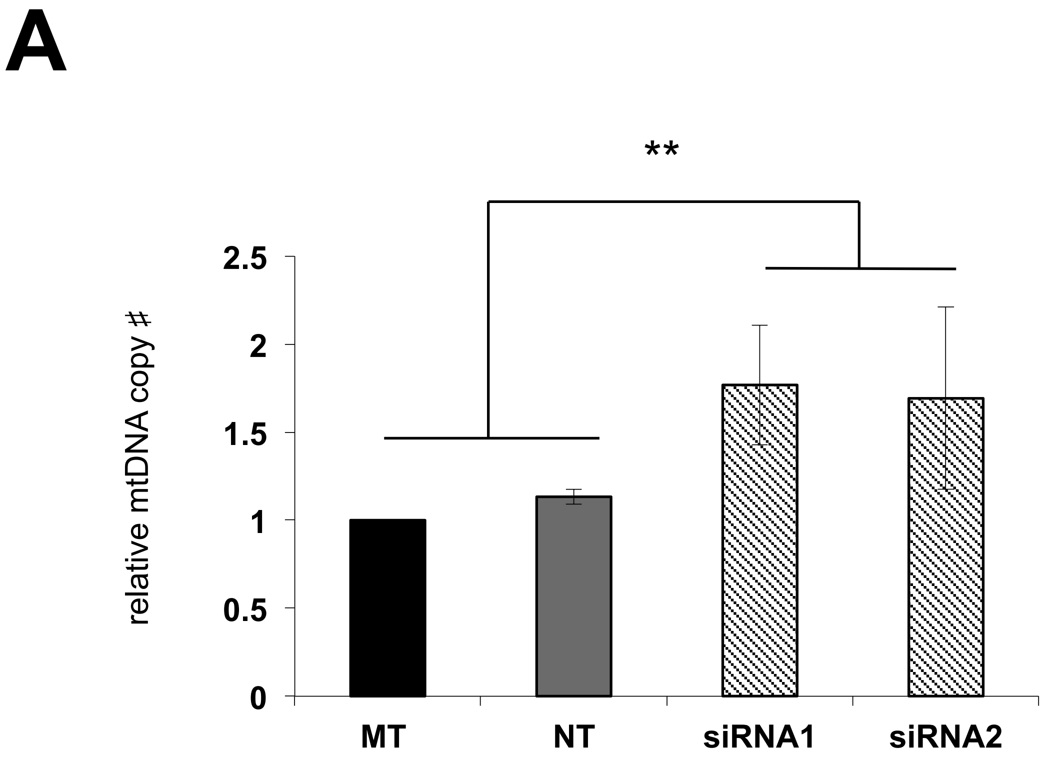

Figure S2 (page 1 of 2)

B

Mitochondrial RNA levels are little affected by MPV17L2 gene silencing

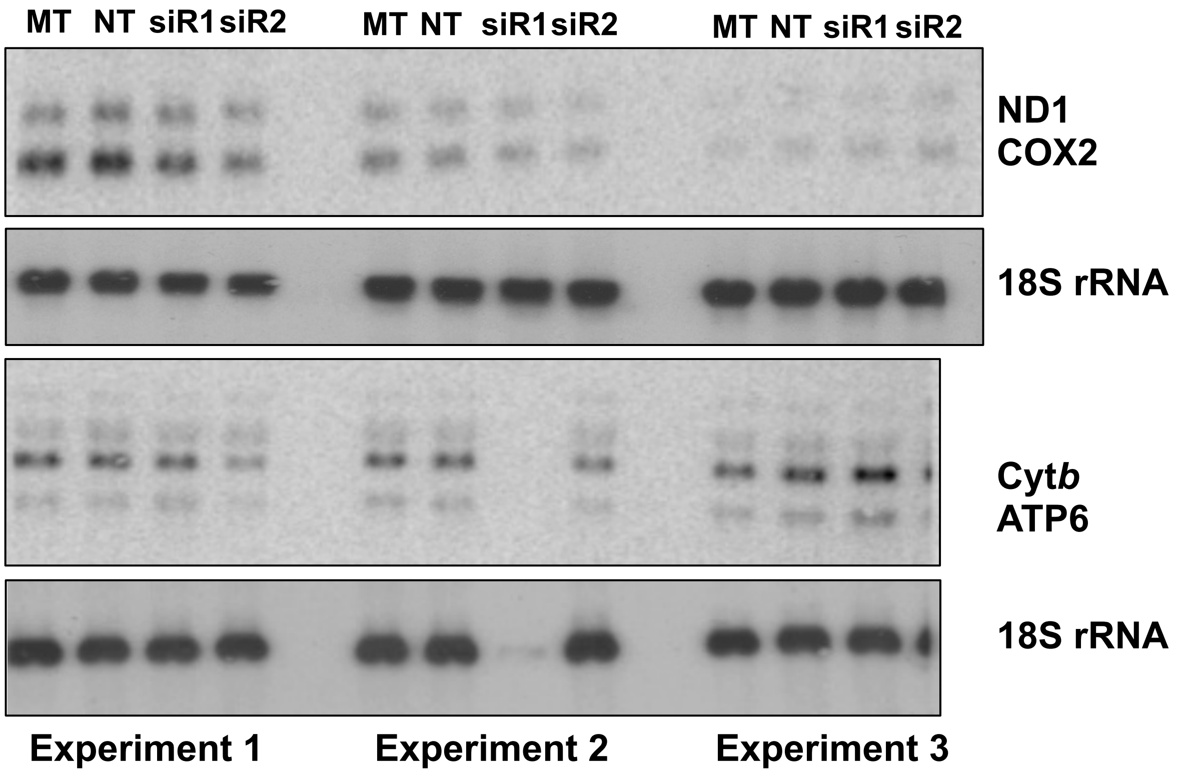

Recombinant SYM1 is not associated with the mitochondrial ribosome

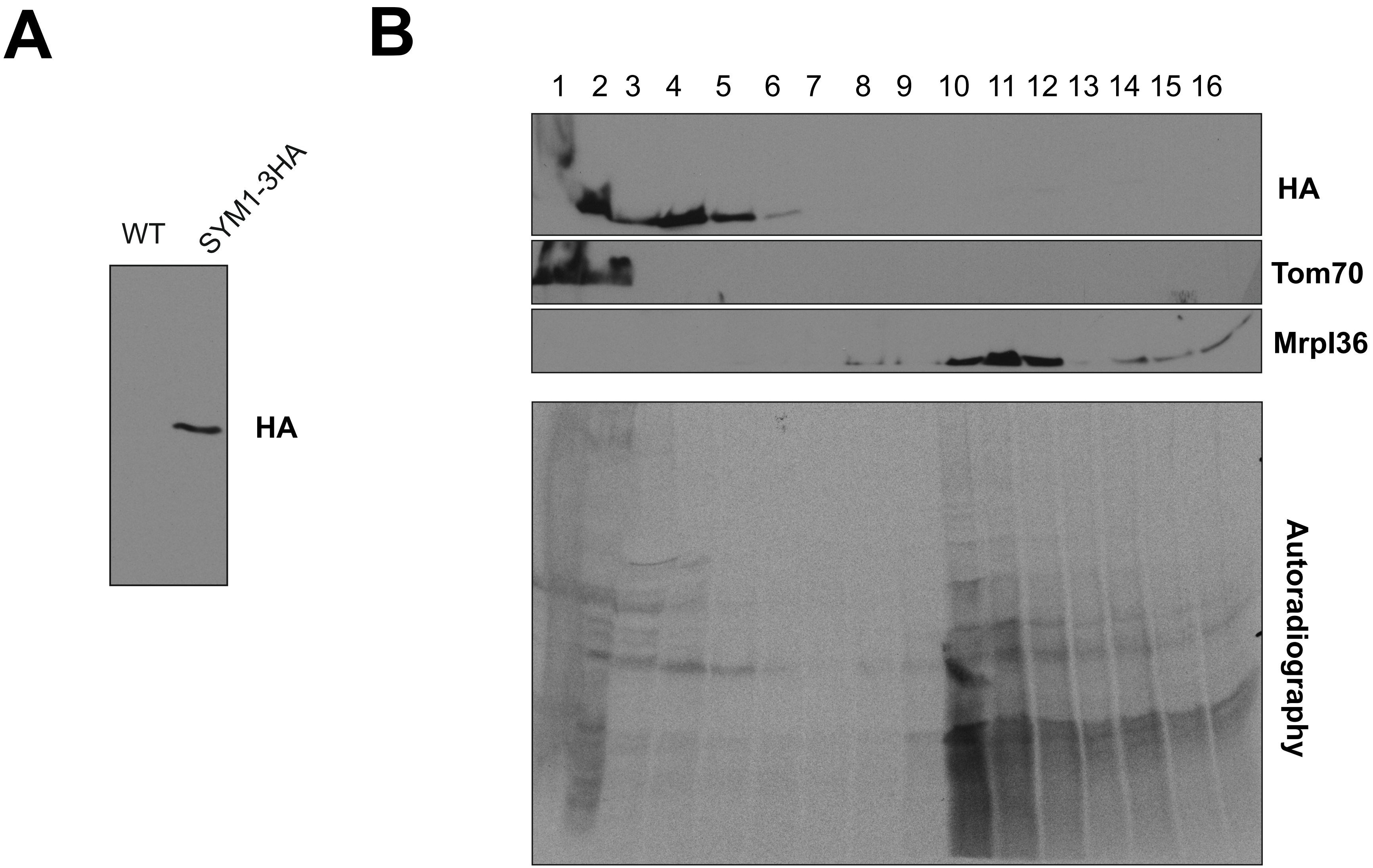

Figure S3
